# Specific Premature Ventricular Complex Characteristics in Women: Insights from a Patient Cohort

**DOI:** 10.3390/jcdd12050181

**Published:** 2025-05-13

**Authors:** Ștefan Ailoaei, Laurențiu Șorodoc, Carina Ureche, Nicolae Sîtari, Alexandr Ceasovschih, Mihaela Grecu, Radu Andy Sascău, Cristian Stătescu

**Affiliations:** 1 Department of Cardiology, Institute for Cardiovascular Diseases “Prof. Dr. George I. M. Georgescu”, 700503 Iași, Romania; stefan.ailoaei@yahoo.com (Ș.A.); mihaelagrecu@yahoo.com (M.G.); radu.sascau@gmail.com (R.A.S.); cstatescu@gmail.com (C.S.); 2 Internal Medicine Department, University of Medicine and Pharmacy “Grigore T. Popa”, 700115 Iași, Romaniaalexandr.ceasovschih@yahoo.com (A.C.); 3 Internal Medicine Clinic, Emergency Clinical County Hospital “Sfantul Spiridon”, 700111 Iași, Romania

**Keywords:** premature ventricular contractions, cardiac magnetic resonance, late gadolinium enhancement, women

## Abstract

Background: Premature ventricular complexes (PVCs) are common arrhythmias that can range from benign to clinically significant. While PVCs have been extensively studied in the general population, gender-specific differences in their characteristics, prevalence, and clinical impact remain underexplored. This study aims to investigate the unique features of PVCs in women and their potential implications for diagnosis and management. Methods: We analyzed a cohort of female patients diagnosed with PVCs, assessing their electrocardiographic patterns, symptomatology, and clinical outcomes. Data were collected from medical records, including Holter monitoring, electrocardiograms (ECGs), and echocardiographic findings. The study also evaluated the association between PVC burden and underlying cardiac conditions. Results: This study analyzed 161 patients (59 females, 91 males) with PVCs, revealing significant sex-based differences. Males were older, had higher BMI, and smoked more, while females experienced more presyncope. ECGs showed greater QRS fragmentation in males. TTE and CMR found males had larger ventricles, lower EF, and more myocardial fibrosis (LGE: 59.34% vs. 37.93%). Patients with LGE were older and had worse clinical outcomes, including higher ICD implantation and hospitalization rates. Despite these structural differences, treatment efficacy was similar across groups. Conclusion: This study highlights key differences in PVC characteristics among women, underscoring the need for gender-specific approaches in clinical evaluation and management. Recognizing these distinctions may aid in early diagnosis, reduce unnecessary interventions, and improve patient outcomes. Further research is warranted to explore the long-term implications of PVCs in women and optimize therapeutic strategies.

## 1. Introduction

Premature ventricular contractions (PVCs) are frequently encountered in clinical settings and often necessitate a comprehensive diagnostic evaluation to rule out underlying cardiomyopathy. While PVCs are common in both men and women, certain characteristics and prevalence rates differ between the two sexes [1,2].

In many cases, no structural heart disease is identified, and the PVCs are considered idiopathic. Over the past decades, cardiac magnetic resonance (CMR) has become the preferred imaging modality for assessing cardiomyopathies, due to its ability to detect subtle myocardial tissue abnormalities and assist in risk stratification [3,4,5,6]. In patients with idiopathic PVCs and a high PVC burden, multiple studies have demonstrated the presence of late gadolinium enhancement (LGE) on CMR. The presence of LGE is indicative of cardiac fibrosis, offering additional diagnostic and prognostic value, as well as guidance for catheter ablation procedures, given the increased risk of adverse cardiac outcomes and arrhythmic events in patients with myocardial scarring [7,8,9].

Focusing on women in PVC research is crucial due to their historical underrepresentation and notable sex-specific differences in arrhythmia characteristics. Women often present with different PVC origins, have distinct electrophysiological profiles, and may respond differently to treatments like ablation. Addressing these differences can improve diagnostic accuracy, personalize therapy, and reduce sex-based disparities in outcomes [1,2,10,11,12,13].

In this context, we conducted a cross-sectional prospective observational study involving a cohort of patients with idiopathic PVCs. Our aim was to analyze PVC characteristics specific to women and compare them with male patients using a real-world database. Additionally, we sought to evaluate whether PVC burden, QRS fragmentation, cardiac fibrosis, response to medication, ablation success, and composite outcomes differ by gender.

## 2. Methods

### 2.1. Patients

We included patients with more than 500 PVCs per 24 h, a normal 12-lead surface electrocardiogram (ECG), and normal findings on 2D transthoracic echocardiography (TTE), with no evidence of significant coronary artery disease as confirmed by either computed tomography coronary angiography or invasive coronary angiography. The primary exclusion criteria were any known structural or ischemic heart disease.

### 2.2. Study Design

We screened 161 patients with premature ventricular contractions (PVCs) who were referred to our center for electrophysiological evaluation between March 2021 and June 2023. Of these, 11 declined participation. Among the remaining patients, we identified 59 eligible women for inclusion and concurrently selected a comparison group of 91 men. All patients underwent follow-up over a 30-month period, with TTE and 24 h Holter monitoring performed every three months.

**Figure 1:**
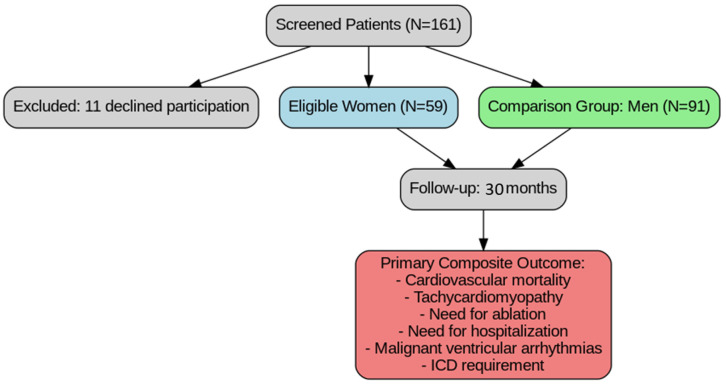
Study design. ICD = implantable cardioverter-defibrillator.

The primary objective was a composite outcome including cardiovascular mortality, tachycardiomyopathy, the need for ablation, the need for hospitalization, malignant ventricular arrhythmias (electrical storm, sustained ventricular tachycardia), and the requirement for an implantable cardioverter-defibrillator (ICD) (Figure 1).

### 2.3. Data Collection and Variables

All baseline clinical data were collected at the time of study enrollment, including information on smoking status, family history of sudden cardiac death (SCD), history of unexplained syncope, arrhythmia-related symptoms, prescription of antiarrhythmic medications, and medical history of hypertension, diabetes, and dyslipidemia. Additionally, body weight, height, and body surface area were measured to calculate the body mass index (BMI).

The baseline 12-lead ECGs were recorded using the Philips PageWriter TC50 electrocardiograph (Böblingen, Germany) and analyzed by experienced operators with a minimum of three years of expertise. Various ECG parameters were assessed, including the native QRS axis, the presence of native QRS fragmentation, native QRS width, PVC QRS width, PVC QRS axis, and PVC coupling interval. Multifocal PVCs were defined as the presence of more than two distinct QRS morphologies.

All patients underwent 24 h monitoring using a 12-channel Holter ECG (Cardiospy EC-12H, Labtech, Hungary), with recordings analyzed by experienced operators with a minimum of three years of expertise. The total number of PVCs and PVC burden were quantified individually for each patient. Additionally, multifocal PVCs were defined as the presence of more than two distinct QRS morphologies observed during Holter monitoring.

TTE was performed at baseline by a single observer with a minimum of three years of experience using the General Electric Vivid S70N Ultra Edition (Boston, MA, USA), following the guidelines of the American Society of Echocardiography and the European Association of Cardiovascular Imaging. A comprehensive assessment was conducted for all patients, evaluating cardiac anatomy and functional parameters included left ventricular ejection fraction (LVEF), measured using Simpson’s biplane method, and right ventricular fractional area change (RVFAC) (2).

All patients underwent comprehensive CMR imaging using a standardized protocol on a 1.5 T platform (Siemens Magnetom Altea 1.5T, München, Germany). The imaging protocol included native acquisitions, consisting of (i) long- and short-axis cine sequences and (ii) tissue mapping (native T1, native T2, and short tau inversion recovery [STIR]).

Ten minutes after the intravenous administration of gadolinium contrast (0.1 mmol/kg Gadovist, Bayer Healthcare, Berlin, Germany), LGE images were acquired in three long-axis views and a complete short-axis stack. Additional dark-blood LGE images were obtained when necessary, particularly in cases where the presence of a subendocardial scar was uncertain.

Image processing and analysis were performed by two experienced operators using the CVI 42 software (Circle Cardiovascular Imaging, Calgary, AB, Canada). Cardiac volumes and mass were assessed by delineating the endocardial and epicardial borders in short-axis cine images (for the ventricles) and long-axis cine images (for the atria) at the end of systole and diastole, with trabeculations excluded from the analysis. All measurements were indexed to body surface area.

LGE was defined as the presence of visual myocardial enhancement in at least one segment, confirmed in either two perpendicular planes or in both bright and black blood images. Subendocardial enhancement with a coronary distribution was classified as ischemic LGE, while all other patterns of fibrosis were categorized as non-ischemic. Fibrosis at the RV insertion points of the interventricular septum was not reported. The LGE percentage for each patient was calculated as the sum of hyperenhanced regions with a signal intensity greater than +5 standard deviations above the normal remote myocardium, divided by the total LV myocardial mass, and expressed as the percentage of the enhanced myocardial mass.

### 2.4. Statistical Analysis

Statistical analysis was conducted using SPSS Statistics 25 software (IBM, Armonk, NY, USA). Continuous variables were presented as either mean ± standard deviation (SD) for normally distributed data or as median with interquartile range (IQR) for non-normally distributed data. Categorical variables were analyzed using the chi-square test or Fisher’s exact test, as appropriate. Comparisons between the means of two independent groups for continuous variables were performed using the independent *t*-test. To assess correlations between variables, Pearson’s correlation coefficient was employed. Kaplan–Meier survival analysis was used to evaluate the time to reaching the composite endpoint.

## 3. Results

### 3.1. Study Population

After screening 161 patients with PVCs we included 59 females and 91 males for further analysis (Table 1). Significant sex-related differences emerged in baseline characteristics: males were significantly older (54.21 ± 15.68 vs. 49.27 ± 15.71 years, *p* = 0.0488) and had a higher body mass index (29.03 ± 3.66 vs. 26.02 ± 4.68 kg/m^2^, *p* < 0.0001) compared to females. Smoking was notably more prevalent among males (39.56% vs. 15.25%, *p* = 0.0028), while no statistically significant differences were found in the prevalence of diabetes mellitus or in family history of sudden cardiac death.

Clinical symptomatology also showed some variation. Presyncope was significantly more common in females (11.86% vs. 2.20%, *p* = 0.0289), suggesting a potential sex-based difference in autonomic or hemodynamic response. Although more males were asymptomatic (9.89% vs. 3.39%), this was not statistically significant (*p* = 0.2020). Rates of syncope were similar between groups, and palpitations were the most frequently reported symptom in both sexes, without significant differences (91.53% in females vs. 87.91% in males, *p* = 0.6675).

Differences were also seen in treatment approaches. Class IC antiarrhythmic drugs were significantly more commonly prescribed to females (66.1% vs. 26.37%, *p* < 0.0001), while beta-blockers were used more frequently in males (81.31% vs. 66.1%, *p* = 0.0346). Although amiodarone was slightly more commonly used in males and sotalol usage was similar between groups, these differences were not statistically significant. Overall, the data highlight meaningful sex-based distinctions in clinical characteristics, symptom presentation, and therapeutic strategies among patients with PVCs.

### 3.2. Electrocardiographic and PVC Characteristics

The analysis of ECG parameters and PVC characteristics revealed several sex-related differences, though most were not statistically significant (Table 2). QRS fragmentation on native complexes was more common in males (18.68%) than females (13.79%), and baseline ECG microvoltage was similar between groups. PVC burden was slightly higher in females (17.20%) compared to males (15.80%), and the initial R wave duration of PVCs was marginally longer in males, but neither difference reached statistical significance. QRS duration of PVCs was nearly identical across sexes.

Polyfocal PVCs occurred at similar rates in both groups. Notably, QRS fragmentation on PVCs was significantly more frequent in males (47.25%) than in females (27.12%, *p* = 0.0217), suggesting possible sex-related differences in ventricular conduction. Regarding PVC morphology, the LBBB pattern with an inferior axis was most common in both sexes. However, males showed a significantly higher frequency of LBBB with a superior axis (13.19% vs. 3.39%, *p* = 0.0481). Other axis and bundle branch block patterns did not differ significantly between sexes.

### 3.3. TTE and CMR Characteristics of the Study Population

TTE and CMR data demonstrated notable sex-related differences in both cardiac structure and function among patients with PVCs. As shown by TTE, males had significantly larger LV end-diastolic volumes (164.60 mL vs. 138.03 mL, *p* = 0.0041), lower EF (49.65% vs. 54.84%, *p* = 0.0009), and reduced global longitudinal strain (17.20% vs. 17.94%, *p* = 0.0318) compared to females, indicating more impaired systolic function. Males also had larger left atrial volumes and lower RV FAC suggesting broader chamber enlargement and reduced RV function (Table 3).

CMR findings were consistent with these differences: males showed significantly larger LV end-diastolic (50.34 mm vs. 45.12 mm, *p* = 0.0021) and end-systolic diameters, increased RV end-diastolic volumes (140.76 mL vs. 125.34 mL, *p* = 0.0298), and larger atrial areas. Both left and RVEF were significantly lower in males. Notably, the presence of LGE—a marker of myocardial fibrosis—was significantly higher in males (59.34%) compared to females (37.93%, *p* = 0.0173), indicating a greater degree of underlying structural myocardial abnormalities in men. These findings highlight key sex differences in cardiac remodeling and fibrosis in patients with frequent PVCs (Table 4).

### 3.4. Correlation of PVC Morphology, Clinical Characteristics, and CMR Scar Presence

**Figure 2:**
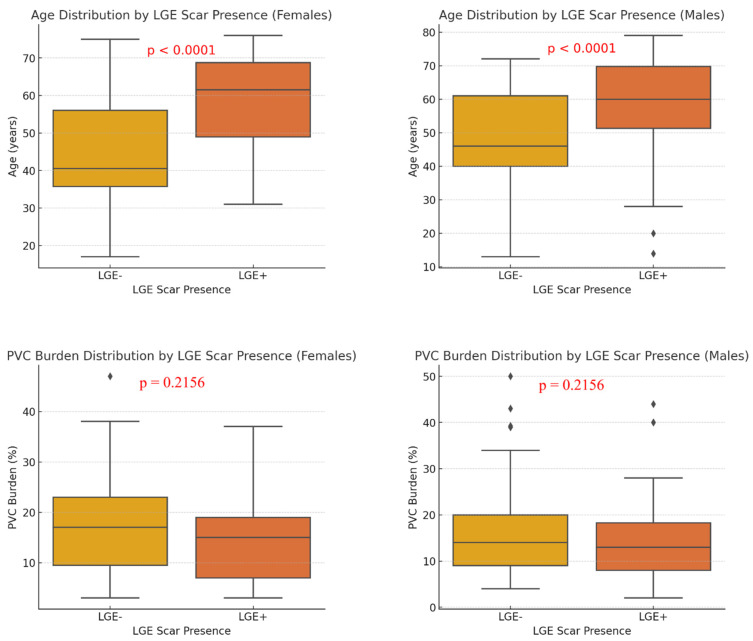
Age and PVC burden distribution by LGE scar presence. LGE—late gadolinium enhancement, PVC—premature ventricular contraction. This figure illustrates the distribution of age (top row) and PVC burden (bottom row) based on the presence of late gadolinium enhancement (LGE) in females (left column) and males (right column). The presence of LGE is significantly associated with older age in both sexes (*p* < 0.0001), while PVC burden does not show a significant difference between LGE+ and LGE− groups (*p* = 0.2156).

Analysis of patient characteristics based on the presence of late gadolinium enhancement (LGE) revealed a significant age difference (Figure 2). Patients with LGE (LGE+) were notably older than those without LGE (LGE−) (56.23 ± 12.45 years vs. 45.89 ± 10.76 years, *p* < 0.0001), indicating a possible association between myocardial fibrosis and advancing age. In contrast, PVC burden was comparable between groups, with LGE+ patients showing a slightly higher burden (18.34 ± 7.65% vs. 17.12 ± 6.98%), though this difference was not statistically significant (*p* = 0.2156).

**Figure 3:**
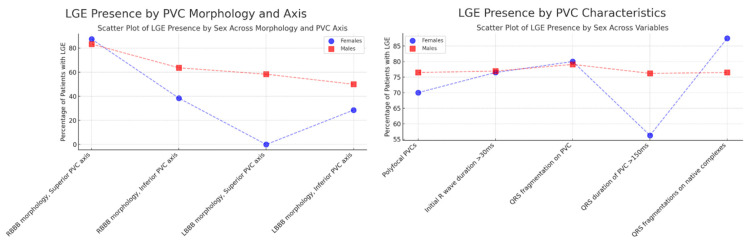
LGE presence by PVC morphology, axis, and characteristics. LGE—late gadolinium enhancement, PVC—premature ventricular contraction, RBBB—right bundle branch block, LBBB—left bundle branch block.

Analysis of PVC characteristics in LGE-positive (LGE+) patients revealed several sex-related patterns (Table 5, Figure 3). Polyfocal PVCs were common in both females and males (70.00% vs. 76.47%, *p* = 0.9965), and initial R wave duration > 30 ms was similarly prevalent (76.47% vs. 76.92%, *p* = 0.1359), indicating comparable early depolarization abnormalities. QRS fragmentation on PVCs was slightly more frequent in females (80.00%) than in males (79.07%), reaching statistical significance (*p* = 0.0147), suggesting a potential sex-based difference in ventricular conduction. Males showed a higher frequency of QRS duration > 150 ms (76.19% vs. 56.25%), though not statistically significant (*p* = 0.6696).

QRS fragmentation on native complexes was also slightly more common in females (87.50% vs. 76.47%, *p* = 0.5798). Regarding PVC morphology, RBBB with a superior axis was prevalent in both sexes (87.50% in females vs. 83.33% in males), while LBBB with a superior axis was seen only in males (58.33%). An LBBB with an inferior axis was more frequent in males (50.00% vs. 28.57%, *p* = 0.0828), indicating a possible trend toward sex-related differences in PVC axis distribution.

Regarding the impact of PVC morphology on the need of ablation, among patients with LBBB morphology, 23 (25.27%) males and 14 (23.73%) females required ablation (*p* > 0.05), while for RBBB morphology, the rates were 13 (14.29%) for males and eight (13.56%) for females (*p* > 0.05). For LBBB morphology with inferior axis, ablation was needed in 20 (32.79%) males and 12 (31.58%) females. However, none of these differences were statistically significant.

**Figure 4:**
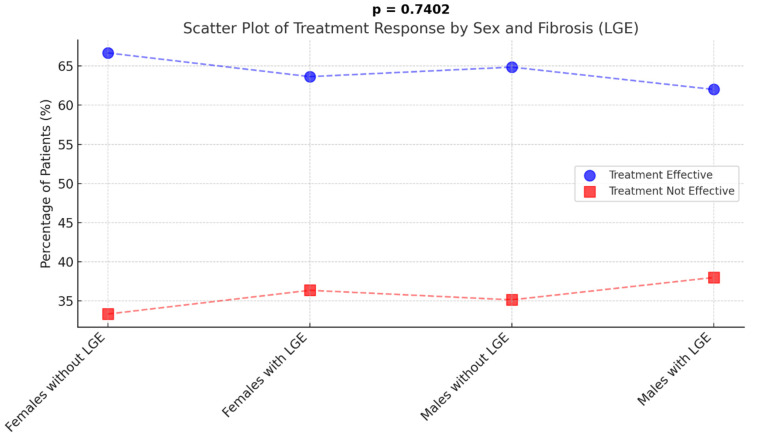
Relationship between LGE presence and treatment efficacy. LGE—late gadolinium enhancement.

The analysis of treatment efficacy in patients with and without LGE showed no significant differences between groups (*p* = 0.7402) (Figure 4). In the female group, 24 out of 36 patients (66.67%) without LGE and 14 out of 22 (63.64%) with LGE responded positively to treatment. Similarly, among males, 24 out of 37 patients (64.86%) without LGE and 31 out of 50 (62.00%) with LGE showed an effective treatment response. These results indicate that the presence of LGE does not appear to influence treatment success, as response rates were similar across all groups.

Looking at the impact of QRS morphology, TTE, and CMR data on the final diagnosis of the patients included in this cohort, among patients with an LBBB morphology with superior axis, three were diagnosed with arrythmogenic cardiomyopathy and one with cardiac sarcoidosis. Additionally, in total, 23 patients (15.3%) of the patients received an additional diagnosis. They all were LGE+ and the distribution of the LGE was important for sustaining the final diagnosis—five were diagnosed with arrythmogenic cardiomyopathy (one female, four males), one with cardiac sarcoidosis (one male), four with mitral valve prolapse and mitral annular disjunction (four females), two with old embolic myocardial infarctions (two males), 10 with non-dilated left ventricular cardiomyopathy (two females, eight males), and one with excessive trabeculation (one male) (Supplementary Figure S1 and Table S1).

### 3.5. Sex-Specific Clinical Outcomes in Patients with and Without LGE

**Figure 5:**
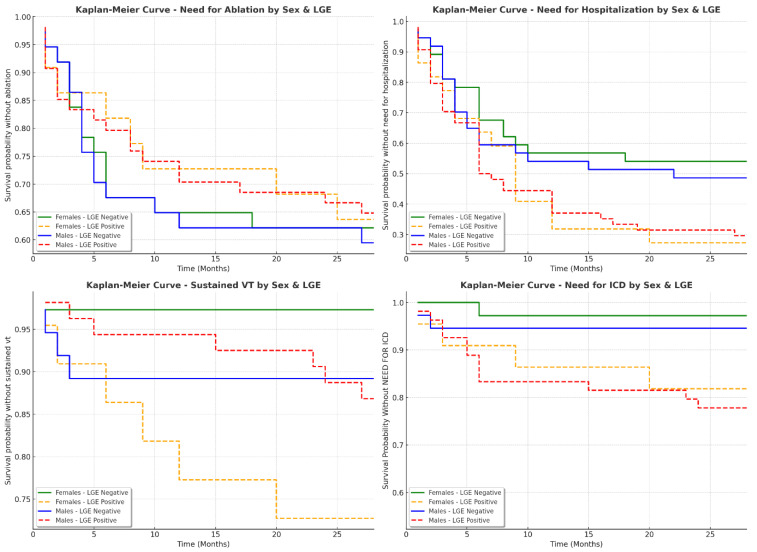
Kaplan–Meier curves for clinical outcomes based on LGE presence and sex. ICD—implantable cardioverter-defibrillator, LGE—late gadolinium enhancement, VT—ventricular tachycardia. Patients with LGE-positive status (especially males) tend to experience these endpoints earlier than their LGE-negative counterparts, suggesting a role of myocardial fibrosis in arrhythmic risk progression.

**Figure 6:**
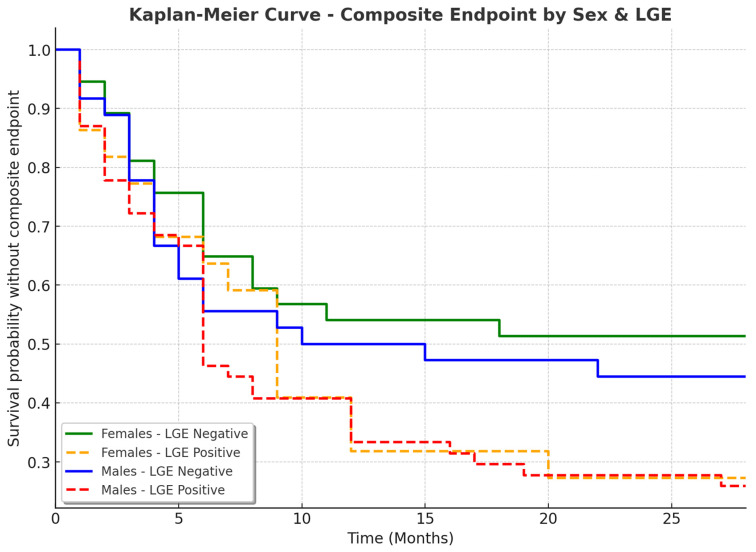
Kaplan–Meier Curve for time to composite endpoint based on LGE presence and sex. LGE—late gadolinium enhancement. These findings reinforce the impact of LGE as a prognostic marker for worse clinical outcomes in both sexes.

The analysis of clinical outcomes in patients based on LGE presence revealed important differences, particularly in the need for an ICD and hospitalization rates (Table 6, Figure 5). A composite endpoint (cardiovascular mortality, tachycardiomyopathy, the need for ablation, the need for hospitalization, malignant ventricular arrhythmias (electrical storm, sustained ventricular tachycardia), and the requirement for an ICD) was reached more frequently in patients with LGE. Among females, 16 out of 22 (72.73%) with LGE reached the composite endpoint, compared to 17 out of 36 (47.22%) of those without LGE (Figure 6). Similarly, in males, 40 out of 54 (74.07%) with LGE reached the endpoint, compared to 20 out of 37 (54.05%) without LGE (*p* = 0.0520). The need for an ICD was significantly higher in LGE-positive patients (*p* = 0.0043). In the female group, only one out of 36 (2.78%) without LGE required an ICD, compared to five out of 22 (22.73%) of those with LGE. Among males, two out of 37 (5.56%) without LGE required an ICD, whereas 15 out of 54 (28.85%) of those with LGE needed one. Hospitalization rates followed a similar pattern, with higher frequencies in LGE-positive patients (*p* = 0.0528). In females, 16 out of 22 (72.73%) with LGE required hospitalization compared to 16 out of 36 (44.44%) without LGE. In males, 38 out of 54 (70.37%) of LGE-positive patients required hospitalization versus 19 out of 37 (51.35%) of those without LGE.

The need for catheter ablation was similar across groups (*p* = 0.7395). In females, eight out of 22 (36.36%) with LGE and 13 out of 36 (36.11%) without LGE underwent ablation. In males, 19 out of 54 (35.19%) with LGE and 15 out of 37 (40.54%) without LGE required ablation. Ventricular tachycardia (VT) was more frequent in LGE-positive patients, although the difference was not statistically significant (*p* = 0.0736). Among females, six out of 22 (28.57%) with LGE had documented VT compared to one out of 36 (2.78%) without LGE. In males, seven out of 54 (12.96%) with LGE experienced VT compared to four out of 37 (10.81%) without LGE. These findings suggest that LGE presence is associated with worse clinical outcomes, including a higher likelihood of ICD implantation, hospitalization, and reaching a composite endpoint. However, the need for ablation remained similar regardless of LGE status. This highlights the potential role of myocardial fibrosis in arrhythmic risk stratification and the importance of cardiac MRI in guiding patient management.

## 4. Discussion

Our study highlights significant differences in the characteristics of PVCs between men and women. Female patients had a lower prevalence of QRS fragmentation on PVCs but exhibited a higher frequency of presyncope compared to males. Additionally, women showed a lower PVC burden, a slightly higher LVEF, and fewer instances of LGE compared to men. These findings suggest that women may have a different arrhythmic substrate, which could have implications for both diagnosis and management.

These findings support the notion that PVC management should be tailored based on sex-specific differences. The higher prevalence of LGE in men suggests that male patients may require more aggressive risk stratification, including earlier consideration of ICD therapy in those with high PVC burden and myocardial fibrosis [4,9,14]. Conversely, women with PVCs may respond better to pharmacological therapy, particularly class IC antiarrhythmic drugs (IC AADs), which were more frequently prescribed in female patients in our study.

Recent studies have highlighted significant sex-related differences in patients with idiopathic premature ventricular contractions (PVCs), particularly in relation to myocardial scarring detected by late gadolinium enhancement (LGE) on cardiac magnetic resonance (CMR) imaging [2,11,12]. A study from our group involving 94 patients with frequent idiopathic PVCs and structurally normal hearts found that 71.23% had myocardial scarring detected by CMR. Male sex, age over 50, and PVCs with a left bundle branch block (LBBB) inferior axis pattern were associated with the presence of LGE, with age and the LBBB inferior axis pattern remaining independent predictors in multivariate analysis. However, LGE was not associated with the need for ablation in this cohort [7]. Similarly, a large retrospective study of 1185 patients undergoing PVC ablation identified male sex as an independent predictor for the development of PVC-induced cardiomyopathy (PIC) [15].

Supporting these findings, a community-based study in Makassar City, Indonesia, which analyzed 8847 ECGs, identified PVCs in 1.1% of the population. Interestingly, 52% of these cases were in women, suggesting a slightly higher prevalence in females. However, men exhibited PVCs with longer QRS duration and a higher incidence of right bundle branch block (RBBB) morphology—features associated with an elevated risk of developing cardiomyopathy [16].

In addition to structural and electrocardiographic differences, hormonal influences may also contribute to sex-related variation in PVCs. One study comparing male patients with idiopathic outflow tract ventricular arrhythmias (IOTVAs) to healthy controls found significantly lower estradiol levels in the IOTVA group. Moreover, there was a negative correlation between estradiol levels and PVC burden, suggesting that reduced estradiol may be linked to the occurrence of IOTVAs in adult males [17]. Although the underlying mechanisms remain unclear, the influence of sex hormones—particularly their effects on cardiac ion channels—has been proposed as a contributing factor [13,15,17,18,19,20].

Several limitations should be acknowledged in our study. The retrospective design introduces the potential for selection bias, as only patients referred for electrophysiological evaluation were included, possibly excluding individuals with milder PVC burden. Another limitation is the relatively short follow-up duration, with patients monitored for only 30 months. A longer follow-up would be necessary to fully assess the long-term risk of arrhythmic events and the progression to PIC.

Further research is necessary to build upon our findings. A prospective, longitudinal cohort study would help confirm the results and minimize the biases associated with retrospective data collection. Future clinical trials should explore the benefits of personalized treatment strategies based on sex-specific characteristics—such as differences in arrhythmic substrate, presence of myocardial fibrosis, symptom presentation, and response to specific antiarrhythmic therapies—to determine whether these tailored approaches lead to improved arrhythmia management and better patient outcomes [21,22,23,24,25,26].

## 5. Conclusions

Our study highlights key sex-based differences in PVC characteristics and underlying myocardial substrate. Women had lower PVC burden, less myocardial fibrosis, and presented with more pronounced symptoms, while men showed more QRS fragmentation and higher rates of LGE. These findings had an important impact on the management strategy and the final diagnosis, suggesting the need for sex-specific management—potentially more aggressive risk stratification in men and a greater role for pharmacological therapy in women. Further prospective studies with longer follow-up are needed to confirm these observations and guide tailored treatment strategies.

## Figures and Tables

**Table 1:** Baseline characteristics of the study population.

	Female (n, %)	Male (n, %)	*p*-Value
***General Characteristics***			
Age (mean ± SD)	49.27 ± 15.71	54.21 ± 15.68	0.0488
BMI (kg/m^2^)	26.02 ± 4.68	29.03 ± 3.66	<**0.0001**
Family history of SCD	4 (6.78%)	4 (4.40%)	0.7124
Smoker	9 (15.25%)	36 (39.56%)	**0.0028**
Diabetes mellitus	9 (15.25%)	22 (24.18%)	0.2662
Sedentary	7 (11.86%)	13 (14.28%)	0.002
***Symptoms***			
Asymptomatic	2 (3.39%)	9 (9.89%)	0.202
Syncope	5 (8.47%)	12 (13.19%)	0.5315
Presyncope	7 (11.86%)	2 (2.20%)	**0.0289**
Palpitations	54 (91.53%)	80 (87.91%)	0.6675
***Medical Therapy***			
IC AADs	39 (66.1%)	24 (26.37%)	<**0.0001**
Amiodarone	15 (25.42%)	32 (35.16%)	0.2089
Betablockers	39 (66.1%)	74 (81.31%)	**0.0346**
Sotalol	3 (5.08%)	1 (1.09%)	0.1388

Note: Values n (%). Abbreviations: BMI—body mass index, SCD—sudden cardiac death, IC AADs—class IC antiarrhythmic drugs.

**Table 2:** Electrocardiographic and PVC characteristics of the study population.

	Female (Mean ± SD or n, %)	Male (Mean ± SD or n, %)	*p*-Value
QRS fragmentations on native complexes	8 (13.79%)	17 (18.68%)	0.6088
Baseline ECG microvoltage	6 (10.34%)	6 (6.59%)	0.6088
PVC burden, %	17.20 ± 10.02	15.80 ± 10.53	0.2378
Initial R wave duration on PVC, ms	30.97 ± 14.87	35.13 ± 17.30	0.0984
QRS duration of PVC, ms	144.66 ± 17.41	144.37 ± 16.44	0.9289
Polyfocal PVCs	10 (16.95%)	17 (18.68%)	0.9584
QRS fragmentation on PVC	16 (27.12%)	43 (47.25%)	**0.0217**
LBBB inferior axis	35/59 (59.32%)	48/91 (52.75%)	0.4532
LBBB superior axis	2/59 (3.39%)	12/91 (13.19%)	**0.0481**
RBBB inferior axis	13/59 (22.03%)	11/91 (12.09%)	0.1173
RBBB superior axis	8/59 (13.56%)	18/91 (19.78%)	0.3415

Note: Values are mean ± SD and n (%). Abbreviations: ECG—electrocardiogram, LBBB—left bundle branch block, PVC—premature ventricular contraction, RBBB—right bundle branch block.

**Table 3:** TTE parameters of the study population.

	Female (Mean ± SD)	Male (Mean ± SD)	*p*-Value
LV EDV, mL	82 ± 12.51	110 ± 18.4	**0.0041**
LV EDV indexed, mL/m^2^	47.3 ± 7.23	59.45 ± 9.94	**0.003**
IVS, mm	10.55 ± 6.93	12.62 ± 17.10	0.3066
EF, %	54.84 ± 11.01	49.65 ± 12.23	**0.0009**
GLS, %	17.94 ± 3.99	17.20 ± 3.33	**0.0318**
LA volume, mL	50.10 ± 25.49	58.50 ± 24.86	<**0.0001**
LA volume indexed, mL/m^2^	28.9 ± 14.73	31.62 ± 13.43	**0.0021**
RV FAC, %	49.39 ± 5.36	47.56 ± 5.42	**0.0169**

Note: Values are mean ± SD. Abbreviations: EF—ejection fraction, GLS—global longitudinal strain, IVS—interventricular septum thickness, LA—left atrium, LV EDV—left ventricular end-diastolic volume, RV FAC—right ventricular fractional area change.

**Table 4:** CMR characteristics of the study population.

Category	Female (Mean ± SD or n, %)	Male (Mean ± SD or n, %)	*p*-Value
EDDLV, mm	45.12 ± 5.76	50.34 ± 6.88	**0.0021**
ESDLV, mm	29.45 ± 6.32	34.21 ± 7.45	**0.0048**
IVS, mm	10.58 ± 2.14	12.67 ± 2.98	**0.0312**
LWT, mm	8.89 ± 1.76	9.45 ± 2.01	0.2156
EDV LV indexed, mL/m^2^	83.76 ± 21.56	87.24 ± 22.7	**0.003**
EDV RV indexed, mL/m^2^	72.45 ± 20.04	76.08 ± 21.19	**0.0298**
RVEF, %	48.12 ± 6.89	44.78 ± 7.34	**0.0412**
LVEF, %	57.23 ± 5.43	53.89 ± 6.01	**0.0185**
LA area, cm^2^	21.34 ± 4.56	24.89 ± 5.67	**0.0376**
RA area, cm^2^	19.45 ± 3.98	21.67 ± 4.32	**0.0461**
LGE scar presence	22 (37.93%)	54 (59.34%)	**0.0173**

Note: Values are mean ± SD and n (%). Abbreviations: EDDLV—end-diastolic diameter of the left ventricle, ESDLV—end-systolic diameter of the left ventricle, EDV RV—end-diastolic volume of the right ventricle, IVS—interventricular septum thickness, LA area—left atrium area, LGE—late gadolinium enhancement, LVEF—left ventricular ejection fraction, LWT—lateral wall thickness, RA—right atrium, RVEF—right ventricular ejection fraction.

**Table 5:** PVC characteristics in LGE+ patients.

	Female with LGE (n, %)	Male with LGE (n, %)	*p*-Value
Polyfocal PVCs	7 (70.00%)	13 (76.47%)	0.9965
Initial R wave duration on PVC > 30 ms	13 (76.47%)	30 (76.92%)	0.1359
QRS fragmentation on PVC	12 (80.00%)	34 (79.07%)	**0.0147**
QRS duration of PVC > 150 ms	9 (56.25%)	16 (76.19%)	0.6696
QRS fragmentations on native complexes	7 (87.50%)	13 (76.47%)	0.5798
RBBB morphology, superior PVC axis	7 (87.50%)	15 (83.33%)	1.0000
RBBB morphology, inferior PVC axis	5 (38.46%)	7 (63.64%)	0.4126
LBBB morphology, superior PVC axis	0 (0.00%)	7 (58.33%)	0.4450
LBBB morphology, inferior PVC axis	10 (28.57%)	24 (50.00%)	0.0828

Note: Values are n (%). Abbreviations: LGE—late gadolinium enhancement, PVC—premature ventricular contraction, RBBB—right bundle branch block, LBBB—left bundle branch block.

**Table 6:** Comparison of clinical outcomes by LGE status and sex.

Variable	Females Without LGE (n, %)	Females with LGE (n, %)	Males Without LGE (n, %)	Males with LGE (n, %)	*p*-Value
**Composite endpoint**	17 (47.22%)	16 (72.73%)	20 (54.05%)	40 (74.07%)	0.0520
**ICD needed**	1 (2.78%)	5 (22.73%)	2 (5.56%)	15 (28.85%)	**0.0043**
**Hospitalization needed**	16 (44.44%)	16 (72.73%)	19 (51.35%)	38 (70.37%)	0.0528
**Ablation needed**	13 (36.11%)	8 (36.36%)	15 (40.54%)	19 (35.19%)	0.7395
**VT present**	1 (2.78%)	6 (28.57%)	4 (10.81%)	7 (12.96%)	0.0736

Note: Values are n (%). Abbreviations: LGE—late gadolinium enhancement, ICD—implantable cardioverter-defibrillator, VT—ventricular tachycardia.

## Data Availability

The original contributions presented in this study are included in the article. Further inquiries can be directed to the corresponding author.

## Supplementary Materials

The following supporting information can be downloaded at: https://www.mdpi.com/article/10.3390/jcdd12050181/s1, Figure S1: Need for Ablation according to PVC morphology and Sex; Table S1: Need for Ablation according to PVC morphology and Sex.

## Abbreviations

BMI	body mass index
CMR	cardiac magnetic resonance
ECG	electrocardiogram
EF	ejection fraction
EDDLV	end-diastolic diameter of the left ventricle
EDV RV	end-diastolic volume of the right ventricle
ESDLV	end-systolic diameter of the left ventricle
FAC	fractional area change
GLS	global longitudinal strain
IC AADs	class IC antiarrhythmic drugs
ICD	implantable cardioverter-defibrillator
IOTVAs	idiopathic outflow tract ventricular arrhythmias
IVS	interventricular septum
LA	left atrium
LBBB	left bundle branch block
LGE	late gadolinium enhancement
LVEF	left ventricular ejection fraction
LWT	lateral wall thickness
PIC	premature ventricular contraction-induced cardiomyopathy
PVCs	premature ventricular contractions
RA	right atrium
RBBB	right bundle branch block
RVEF	right ventricular ejection fraction
RV FAC	right ventricular fractional area change
SCD	sudden cardiac death
TTE	transthoracic echocardiography
VT	ventricular tachycardia
