# Supplementary material for: Specific Premature Ventricular Complex Characteristics in Women: Insights from a Patient Cohort
Source: J Cardiovasc Dev Dis. 2025 May 13;12(5):181. doi: 10.3390/jcdd12050181 (PMC12112119; doi:10.3390/jcdd12050181)
Supplement: Supplementary file 1 [file jcdd-12-00181-s001.zip › jcdd-3588459-supplementary.pdf]

Table S1. Need for Ablation according to PVC morphology and Sex

	Need for ablation		p-value
	Male	Famale	
**LBBB Morphology**	23 (25.27%)	14 (23.73%)	1.0000
**RBBB Morphology**	13 (14.29%)	8 (13.56%)	1.0000
**RBBB Morphology, Superior PVC Axis**	8 (22.86%)	3 (14.29%)	1.0000
**RBBB Morphology, Inferior PVC Axis**	5 (14.29%)	5 (23.81%)	0.9892
**LBBB Morphology, Superior PVC Axis**	3 (4.92%)	2 (5.26%)	0.4936
**LBBB Morphology, Inferior PVC Axis**	20 (32.79%)	12 (31.58%)	0.7041

Note: Values are n (%). Abbreviations: PVC – Premature Ventricular Contractions, RBBB – Right Bundle Branch Block, LBBB – Left Bundle Branch Block.

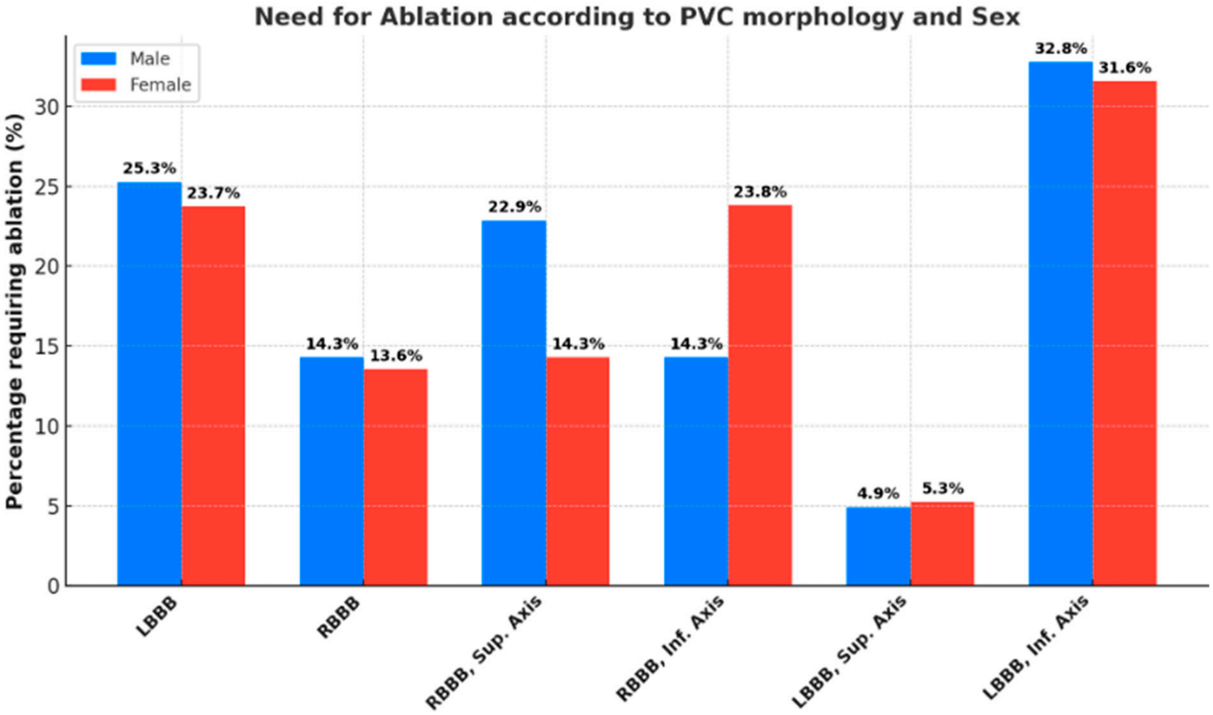

Figure S1. Need for Ablation according to PVC morphology and Sex
